# The oxygen reactivity index indicates disturbed local perfusion regulation after aneurysmal subarachnoid hemorrhage: an observational cohort study

**DOI:** 10.1186/s13054-023-04452-3

**Published:** 2023-06-13

**Authors:** Nick Kastenholz, Murad Megjhani, Catharina Conzen-Dilger, Walid Albanna, Michael Veldeman, Daniel Nametz, Soon Bin Kwon, Henna Schulze-Steinen, Hani Ridwan, Hans Clusmann, Gerrit Alexander Schubert, Soojin Park, Miriam Weiss

**Affiliations:** 1 Department of Neurosurgery, RWTH Aachen University, Aachen, Germany; 2 Program for Hospital and Intensive Care Informatics, Department of Neurology, Columbia University Vagelos College of Physicians and Surgeons, New York City, NY USA; 3 NewYork-Presbyterian Hospital, Columbia University Irving Medical Center, New York City, NY USA; 4 Department of Intensive Care Medicine and Perioperative Care, RWTH Aachen University, Aachen, Germany; 5 Department of Diagnostic and Interventional Neuroradiology, RWTH Aachen University, Aachen, Germany; 6 Department of Neurosurgery, Cantonal Hospital Aarau, Tellstrasse 25, 5001 Aarau, Switzerland; 7 Department of Biomedical Informatics, Columbia University, New York City, NY USA

**Keywords:** Cerebral autoregulation, Vasoreactivity, Brain tissue oxygen, Neuromonitoring, Subarachnoid hemorrhage, Delayed cerebral ischemia

## Abstract

**Background:**

Cerebral autoregulation (CA) can be impaired in patients with delayed cerebral ischemia (DCI) after aneurysmal subarachnoid hemorrhage (aSAH)*.* The Pressure Reactivity Index (PRx, correlation of blood pressure and intracranial pressure) and Oxygen Reactivity Index (ORx, correlation of cerebral perfusion pressure and brain tissue oxygenation, PbtO_2_) are both believed to estimate CA. We hypothesized that CA could be poorer in hypoperfused territories during DCI and that ORx and PRx may not be equally effective in detecting such local variances.

**Methods:**

ORx and PRx were compared daily in 76 patients with aSAH with or without DCI until the time of DCI diagnosis. The ICP/PbtO_2_-probes of DCI patients were retrospectively stratified by being in or outside areas of hypoperfusion via CT perfusion image, resulting in three groups: DCI + /probe + (DCI patients, probe located inside the hypoperfused area), DCI + /probe*− * (probe outside the hypoperfused area), DCI*− * (no DCI).

**Results:**

PRx and ORx were not correlated (*r* = *− *0.01, *p* = 0.56). Mean ORx but not PRx was highest when the probe was located in a hypoperfused area (ORx DCI + /probe + 0.28 ± 0.13 vs. DCI + /probe*− * 0.18 ± 0.15, *p* < 0.05; PRx DCI + /probe + 0.12 ± 0.17 vs. DCI + /probe*− * 0.06 ± 0.20, *p* = 0.35). PRx detected poorer autoregulation during the early phase with relatively higher ICP (days 1–3 after hemorrhage) but did not differentiate the three groups on the following days when ICP was lower on average. ORx was higher in the DCI + /probe + group than in the other two groups from day 3 onward. ORx and PRx did not differ between patients with DCI, whose probe was located elsewhere, and patients without DCI (ORx DCI + /probe*− * 0.18 ± 0.15 vs. DCI*− * 0.20 ± 0.14; *p* = 0.50; PRx DCI + /probe*− * 0.06 ± 0.20 vs. DCI*− * 0.08 ± 0.17, *p* = 0.35).

**Conclusions:**

PRx and ORx are not interchangeable measures of autoregulation, as they likely measure different homeostatic mechanisms. PRx represents the classical cerebrovascular reactivity and might be better suited to detect disturbed autoregulation during phases with moderately elevated ICP. Autoregulation may be poorer in territories affected by DCI. These local perfusion disturbances leading up to DCI may be more readily detected by ORx than PRx. Further research should investigate their robustness to detect DCI and to serve as a basis for autoregulation-targeted treatment after aSAH.

## Background

Delayed cerebral ischemia (DCI) is a major contributor to the high morbidity after aneurysmal subarachnoid hemorrhage (aSAH) [1]. The pathophysiology behind this secondary complication has not been unraveled completely, but besides large- and microvessel vasospasm, neuroinflammation with breakdown of the blood brain barrier, cortical spreading depolarizations or other reasons, impaired cerebral autoregulation (CA) may play an important role in the development of DCI [2–5]. CA is the intrinsic capacity of cerebral vessels to maintain a constant cerebral blood flow (CBF) through myogenic adjustment of vessel tone despite fluctuations in cerebral perfusion pressure (CPP) [6]. Efforts to continuously measure CA in patients are mostly based on correlating changes of mean arterial blood pressure (MAP) or CPP with changes in CBF or a surrogate thereof [7]. The Pressure Reactivity Index (PRx) as a correlation of MAP and intracranial pressure (ICP) has been validated to provide estimations of CA in patients with traumatic brain injury (TBI) [8, 9], correlating strongly with mortality and overall outcome [10]. Because of these findings, the PRx is commonly used in studies on CA after several neurological injuries [7, 11]. However, in patients with aSAH, ambiguous results have been reported on the association of PRx with patient outcome and the development of DCI [12–15]. Alternatively, the Oxygen Reactivity Index (ORx) has been proposed to measure CA as the correlation coefficient between CPP and partial pressure of brain tissue oxygen (PbtO_2_; as a surrogate of CBF) [16]. Studies on ORx in aSAH patients have also yielded equivocal, yet sometimes promising results with associations of the parameter with patient outcome and DCI development [12, 13, 16, 17].

While bedside measurement of CA used to be primarily a matter of academic interest with limited impact on clinical management, strategies to actively preserve CA after neurological injuries by individual pressure targets are now under investigation [18–20]. The leading initiative investigates patients with traumatic brain injury and utilizes PRx as basis for calculating the optimal target (CPPopt) [18]. It is not yet clear which measurement of CA holds most value as a target after aSAH, particularly considering DCI as a disease-specific complication. DCI and DCI-related hypoperfusion are mostly local phenomena centered in a vascular territory or watershed zone [21]. It is currently unknown whether CA functionality can be poorer in hypoperfused territories during DCI than in other brain tissue and, if so, whether PRx and ORx would be able to discriminate such local disturbances. We hypothesized that PRx and ORx, displaying changes of ICP or PbtO_2_ respectively, may yield different results for the functionality of CA in areas that are affected by DCI. Potential differences could depend on the localization and extent of CA impairment in relation to the location of the probe measuring ICP or PbtO_2_. Understanding differences (or lack thereof) in the behavior of CA indices would be important for the design of autoregulation-targeted treatment for the prevention of DCI in patients with aSAH. The aim of this study was therefore an assessment of the physiological development and correlation of PRx and ORx with DCI-related hypoperfusion.

## Methods

### Study population

**Fig. 1:**
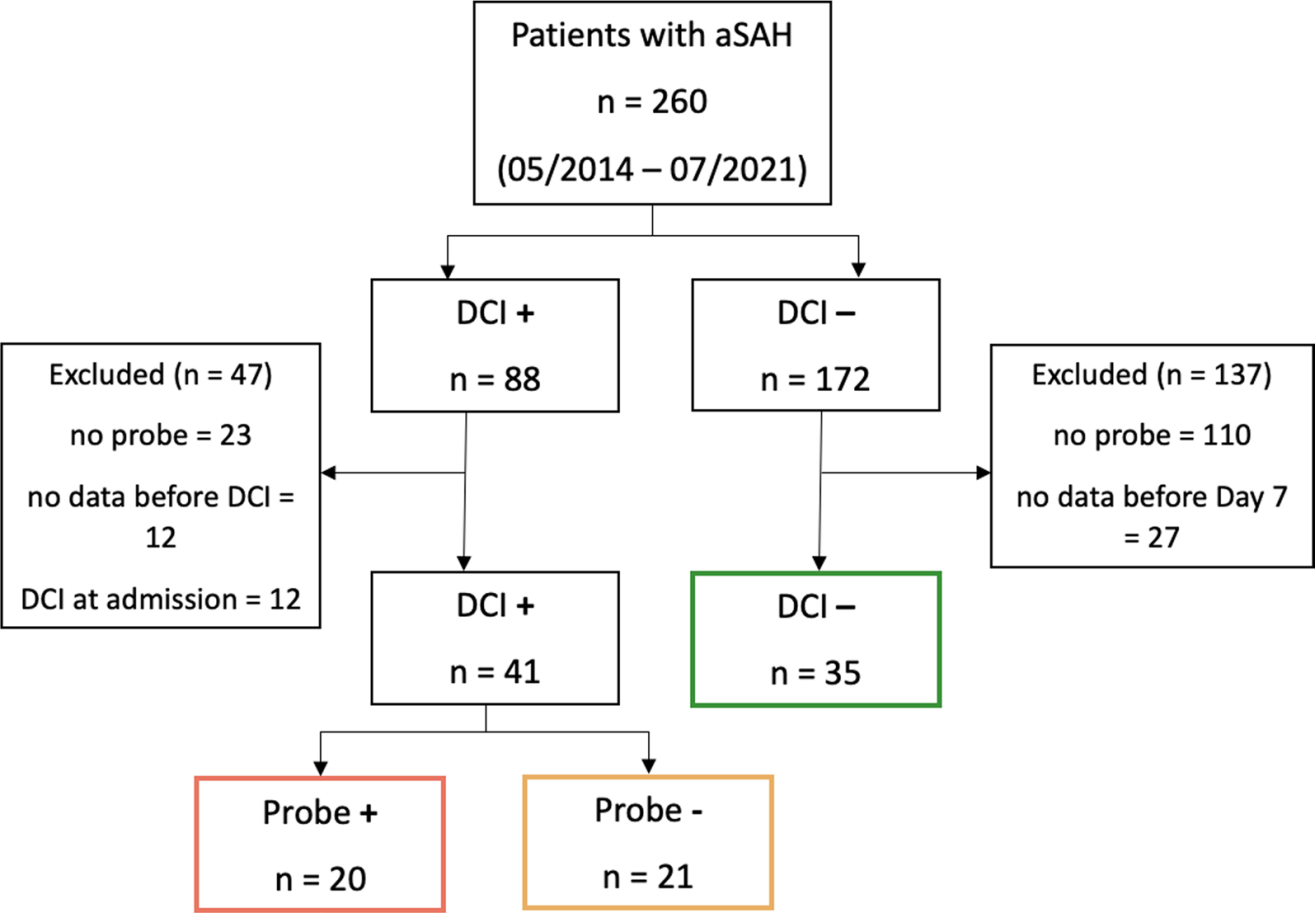
Flow chart of enrollment. Colors indicate group allocation and are standard for the following figures. Two patients were included in the DCI + /probe- group, whose diagnosis of DCI was solely based on clinical symptoms without a perfusion deficit on CT perfusion. aSAH = aneurysmal subarachnoid hemorrhage, DCI = delayed cerebral ischemia, probe + : ICP/PbtO_2_-probe in an area of hypoperfusion, probe-: probe not in area of hypoperfusion

Patients with aSAH were enrolled into a prospective databank at a tertiary institution from May 2014 to July 2021. Informed consent was obtained from the patient, or their legal representatives and data collection were approved by the local ethics committee (EK 062/14). Patients were included for this analysis if they were ≥ 18 years of age, with confirmed aSAH on admission CTA and DSA, and if ORx and PRx data were available for ≥ 12 simultaneous hours before the diagnosis of DCI (Fig. 1). DCI occurred on average 7 days after hemorrhage. From patients without DCI, ≥ 12 h of data were required before day 7 to ensure comparable data time frames between patients with and without DCI. Reporting was aligned to the STrengthening the Reporting of OBservational studies in Epidemiology (STROBE) criteria for observational studies [22].

### Treatment algorithm

Our detailed standard operating procedure has been previously described elsewhere [23]. Patients were closely monitored in a neurocritical care unit following occlusion of the aneurysm via surgical clipping or endovascular coiling (or less commonly, with a flow diverter or WEB device). Invasive neuromonitoring was considered in comatose and/or analgosedated patients who could not be assessed clinically, or in awake patients who were considered to be at high risk of DCI (Hunt and Hess grade 3–5, modified Fisher grade (mFS) 3–4) if no contraindications (coagulation disorders or early predicted mortality) were present. A combined ICP/PbtO_2_-probe (Neurovent PTO, Raumedic, Helmbrechts, Germany) was inserted unilaterally into the frontal watershed zone between anterior and middle cerebral artery territories of the hemisphere where the risk of DCI was expected to be higher (hemisphere carrying the aneurysm or higher blood load or right side in case of midline aneurysm).

### Delayed cerebral ischemia

DCI was defined according to Vergouwen et al. [24]: new focal neurological deficit or decrease in Glasgow Coma Scale ≥ 2 for a duration ≥ 1 h or reversible after treatment and not ascribable to other reasons. Cerebral oxygenation crisis (PbtO_2_ < 20 mmHg) or metabolic derangement (assessed by cerebral microdialysis, lactate/pyruvate ratio > 40) were considered warning signs, triggering CT perfusion (CTP) imaging to also diagnose DCI in case territorial or watershed perfusion deficits were observed. Perfusion deficits on CTP were defined as time to drain greater than 10 s and mean transit time greater than 6.7 s [20]. Minor perfusion delays were observed closely but not considered as DCI. Thus, DCI was also diagnosed in patients with no clinical manifestations according to Vergouwen et al., when hypoperfusions were observed on CTP imaging.

### Monitoring and data acquisition

MAP was invasively measured in the femoral or radial artery using a standard pressure transducer. High frequency waveform data of MAP, ICP (100 Hz) and PbtO_2_ (1 Hz) were acquired and stored using the MPR2 logO Datalogger and Datalogger software (Raumedic) or, after July 2018, the Moberg CNS monitor (Component Neuromonitoring System, Moberg Research, Ambler, PA). PbtO_2_ signals from the initial calibration period following probe placement were manually excluded from the analysis. Physiological parameters were averaged over 60 min for final analysis. Clinical outcome was noted as modified Rankin Scale 3 months after discharge and assessed in person or via structured telephone interview.

### Study design

**Fig. 2:**
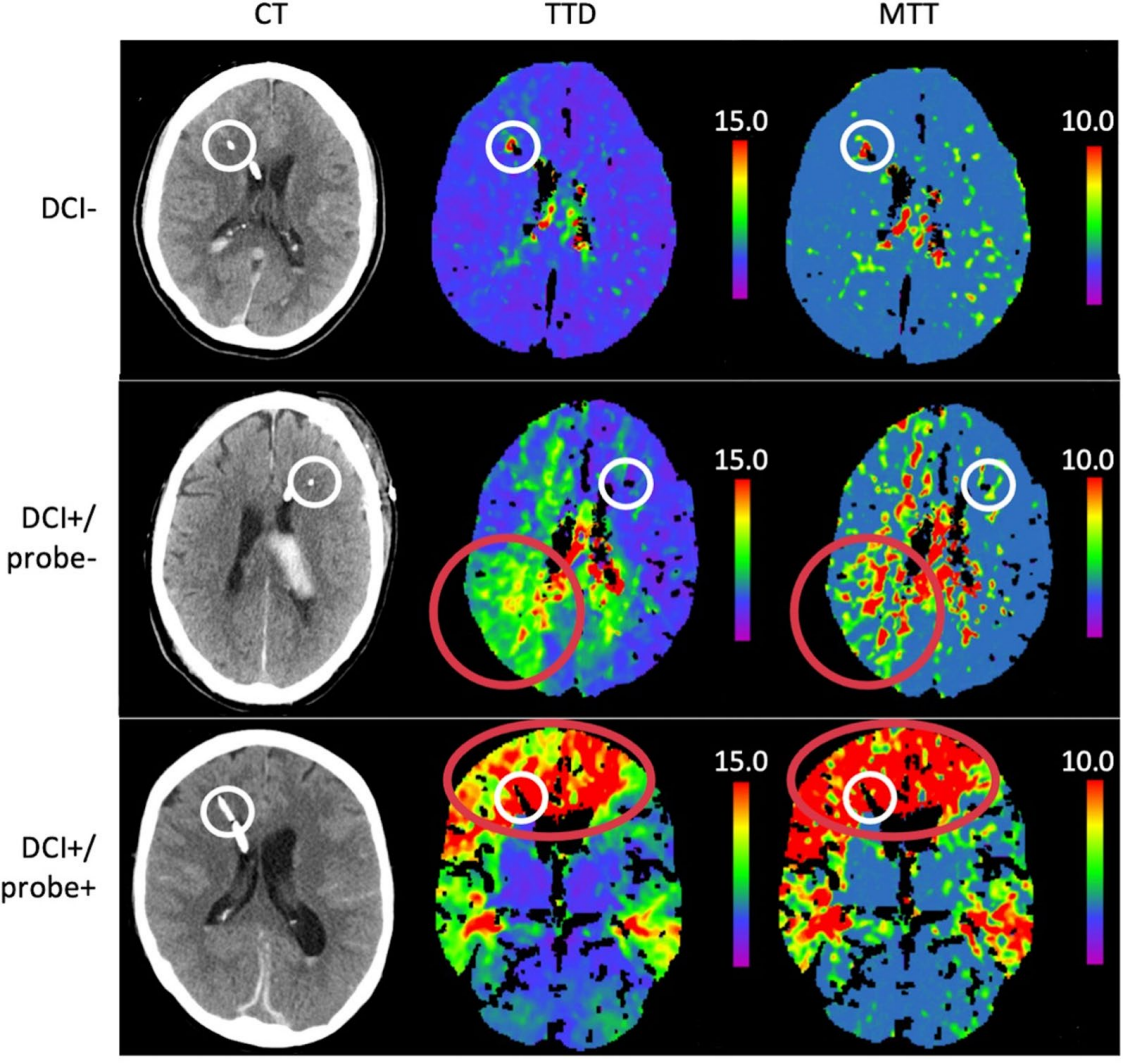
CT perfusion images with time to drain (TTD, seconds) and mean transit time (MTT, seconds) of an example patient from each group. The white circle shows the tip of the combined ICP/PbtO_2_-probe, and the red circle shows the area of hypoperfusion at time of DCI diagnosis

The study population was split into three groups according to the development of DCI and the location of the ICP/PbtO_2_-probe: no DCI, DCI + /probe + and DCI + /probe-, indicating whether the probe was located in or outside of the hypoperfused area during DCI. The CT image (native and perfusion) used to diagnose DCI was the basis for the allocation of the probe (Fig. 2). The probe was considered to be in the hypoperfused area if both the time to drain and mean transit time thresholds were exceeded around the probe tip (DCI + /probe +). If DCI was diagnosed in CT perfusion, but the probe was not located in the area meeting the thresholds, the patient was assigned as DCI + /probe−. Additionally, if DCI was diagnosed clinically without areas of hypoperfusion on CT perfusion, the patient was also assigned as DCI + /probe−. All parameters were synchronized to the time of hemorrhage, to allow the observation of the spontaneous course of PRx and ORx, and compared between the three groups on each postbleeding-day separately for a maximum of seven days. Data were censored after diagnosis of DCI to avoid potential bias through DCI treatment. In patients without DCI, data were censored after day seven (mean time of development of DCI in the DCI + group).

### Calculation of indices

Data processing including artifact removal and calculation of indices was performed using the ICM + multimodal monitoring software (University of Cambridge, Cambridge Enterprise, Cambridge, United Kingdom). PRx was calculated retrospectively as the moving Pearson correlation coefficient of 30 10-s-averaged values of MAP and ICP, resulting in a moving window of 5 min and a new PRx value every minute [8]. PRx may vary between + 1, indicating impaired autoregulation when a passive relationship between MAP and ICP is assumed, and − 1, indicating intact autoregulation as an increase of MAP leads to a decrease of ICP. PRx values > 0.2–0.3 reflect disturbed autoregulation [25]. ORx was defined as the moving correlation coefficient between CPP (MAP—ICP) and PbtO_2_. MAP and ICP were averaged over 30 s and correlated within a 60 min moving window, updated every minute. Thus, every minute a new ORx, which may vary between + 1 and − 1, correspondingly, was calculated from 120 data pairs [26]. All indices were averaged over 60 min.

### Statistical analysis

Statistical tests were conducted within IBM SPSS Statistics 26 (SPSS, Chicago, IL) and Python (Python Software Foundation, https://www.python.org/). Outcome analysis included dichotomization of patients into favorable (mRS 0–3) and unfavorable (mRS 4–6) outcome. Categorical parameters were compared using the Chi-square test and presented as frequencies and proportions, while continuous variables were presented as means and standard deviations, unless stated otherwise. After testing for normality using the Kolmogorov–Smirnov Test, continuous data were compared using the Kruskal–Wallis H test or Student’s t test. Post hoc analysis for pairwise comparisons was made using Dunn’s test with Bonferroni correction to account for multiple comparisons. The calculated indices were compared using Spearman’s Rank-Order Correlation. Statistical significance was assumed at a two-sided *p* value < 0.05.

## Results

### Patient characteristics

The enrollment process is shown in Fig. 1. We included 76 patients for final analysis, whose baseline characteristics are outlined in Table 1. DCI was diagnosed in 41 patients (53.9%). DCI diagnosis was either based on CT perfusion alone (DCI + /probe + : 70%, DCI + /probe-: 66.7%) or CT perfusion and clinical deterioration (DCI + /probe + : 30%, DCI + /probe−: 23.8%). Only two patients who were diagnosed with DCI (4.9%) clinically had no hypoperfusions on CTP and were consequently allocated to the DCI + /probe− group. The ICP/PbtO_2_-probe was located in a hypoperfused area at time of DCI diagnosis in 20 patients (48.8%, DCI + /probe +). On average, 122.4 ± 73.9 h and 117.3 ± 40.0 h of monitoring time were included, respectively (DCI + vs. DCI−, *p* = 0.71). There were no differences in baseline characteristics, except for a greater proportion of mFS 3–4 in patients with DCI (75.6% vs. 42.9%, *p* < 0.005). The average time of DCI was 7.7 ± 3.1 days after hemorrhage.

**Table 1:** Patient characteristics

Parameter	DCI + (*n* = 41)			DCI− (*n* = 35)	*p* value
	Probe + (*n* = 20)	Probe− (*n* = 21)	Total		DCI + versus −
Age (yr)	50.5 ± 12.0	59.0 ± 12.3	54.8 ± 12.8	60.3 ± 11.7	0.06
Female sex, *n* (%)	15 (75.0)	14 (66.7)	29 (70.7)	27 (77.1)	0.53
Hypertension, *n* (%)	7 (35.0)	9 (42.9)	16 (39.0)	14 (40.0)	0.93
Smoking, *n* (%)	4 (20.0)	6 (28.6)	10 (24.4)	5 (23.8)	0.27
HH 4–5, *n* (%)	11 (55.0)	9 (42.9)	20 (48.8)	15 (42.9)	0.61
mFS 3–4, *n* (%)	16 (80.0)	15 (71.4)	31 (75.6)	15 (42.9)	**< 0.005**
Anterior location, *n* (%)	15 (75.0)	16 (76.2)	31 (75.6)	26 (74.3)	0.89
Clipping, *n* (%)	7 (35.0)	11 (52.4)	18 (43.9)	15 (42.9)	0.93
Endovascular, *n* (%)	13 (65.0)	10 (47.6)	23 (56.1)	20 (57.1)	0.93
mRS 4–6, *n* (%)	14 (70.0)	11 (54.4)	25 (58.5)	15 (45.5)	0.18
Time to DCI (h)	176.0 ± 73.5	194.0 ± 77.1	185.7 ± 75.0	-	–
Monitoring time (h)	112.6 ± 72.0	131.8 ± 76.1	122.4 ± 73.9	117.3 ± 40.0	0.71

Overview of baseline patient characteristics for patients with and without development of DCI. The DCI + group was further stratified into patients whose ICP/PbtO_2_-probe was in or out of an area of perfusion deficit at time of DCI diagnosis

*DCI* Delayed cerebral ischemia, *HH* Hunt & Hess grade, *mFS* modified Fisher scale, *mRS* modified Rankin scale (two patients lost to follow-up in the DCI- group), and bold value indicates statistical significance

### Baseline physiological parameters

Mean physiological measurements over the total measuring time including MAP, ICP, CPP and PbtO_2_ were similar in all three groups (Table 2). ICP was significantly higher in DCI + /probe + than DCI + /probe− and DCI− patients during the first 4 days after hemorrhage, most pronounced on day 2 (DCI + /probe + : 12.6 ± 8.7 mmHg vs. DCI + /probe−: 7.4 ± 5.0 mmHg, *p* < 0.0001; Fig. 4C), after which the difference became smaller over time. PbtO_2_ showed an increasing tendency between day 3 and 5 (DCI-: 18.7 ± 12.7 to 25.8 ± 14.3, DCI + /probe + : 18.6 ± 13.0 to 27.2 ± 15.3, DCI + /probe−: 15.6 ± 12.8 to 24.0 ± 13.5 mmHg) in all groups (Fig. 4D). PbtO_2_ was higher in DCI + /probe + patients than in DCI + /probe− patients on several days. CPP increased from day 1 onward (day 1 to day 7; DCI-: 73.0 ± 13.4 to 86.5 ± 14.4, DCI + /probe + : 71.4 ± 14.1 to 85.8 ± 17.8, DCI + /probe-: 79.2 ± 21.4 to 90.8 ± 14.9 mmHg), analogously to MAP (day 1 to day 7; DCI−: 82.3 ± 10.6 to 96.3 ± 14.5, DCI + /probe + : 83.6 ± 12.7 to 94.5 ± 17.6, DCI + /probe−: 85.0 ± 13.8 to 97.4 ± 13.3 mmHg), with no major differences throughout the analyzed period.

**Table 2:** Physiological and autoregulation parameters

Parameter	All	DCI−	DCI + /probe +	DCI + /probe−	*p* value
MAP (mmHg)	92.6 ± 10.5	91.7 ± 10.9	94.2 ± 12.2	92.3 ± 8.3	0.74
ICP (mmHg)	8.8 ± 3.7	9.0 ± 3.6	9.6 ± 4.2	7.6 ± 3.1	0.46
CPP (mmHg)	83.8 ± 10.4	83.0 ± 9.8	84.3 ± 12.6	84.5 ± 9.5	0.83
PbtO_2_ (mmHg)	21.0 ± 10.3	20.9 ± 10.5	21.2 ± 9.3	21.0 ± 11.4	0.91
PRx	0.08 ± 0.18	0.08 ± 0.17	0.12 ± 0.17	0.06 ± 0.2	0.35
ORx	0.21 ± 0.14	0.20 ± 0.14	0.28 ± 0.13	0.18 ± 0.15	**< 0.05**

Bold value indicates statistical significance

Mean arterial pressure (MAP), intracranial pressure (ICP), cerebral perfusion pressure (CPP), partial pressure of brain tissue oxygen (PbtO_2_) and Pressure Reactivity Index (PRx) were comparable when averaged over the total monitoring period (up to DCI or day 7, respectively). ORx was significantly different between the three groups, but not PRx. *p* value given for Kruskal–Wallis H test comparing all groups

### Correlation of PRx and ORx

**Fig. 3:**
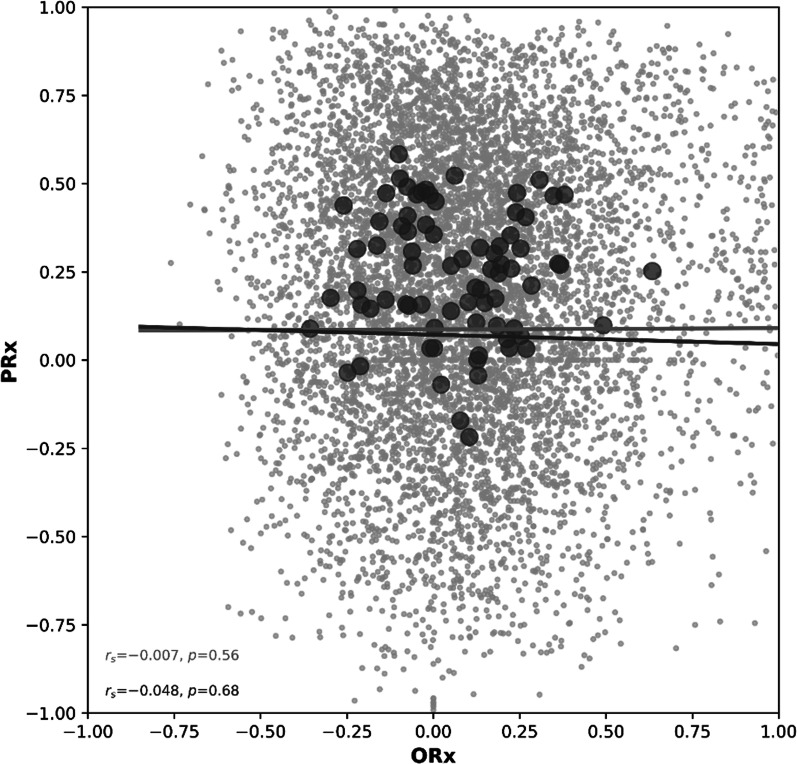
Scatterplot of Pressure Reactivity Index (PRx) versus Oxygen Reactivity Index (ORx). Light gray dots represent hourly data pairs from all patients, and dark gray dots represent one averaged data pair per patient for the entire analyzed period. Spearman’s rank correlation depicts the lack of correlation between PRx and ORx

The correlation coefficients using a single summarized data pair per patient for the entire analyzed period of PRx and ORx of the total cohort were *r* = − 0.007 (*p* = 0.56) and *r* = − 0.048 (*p* = 0.68), indicating no correlation between the two indices (Fig. 3). This did not change when stratified into three DCI groups (DCI− : *r* = − 0.003, *p* = 0.45; DCI + /probe + : *r* = 0.008, *p* = 0.83, DCI + /probe− : *r* = −0.032, *p* = 0.10).

### Temporal development of PRx and ORx

**Fig. 4:**
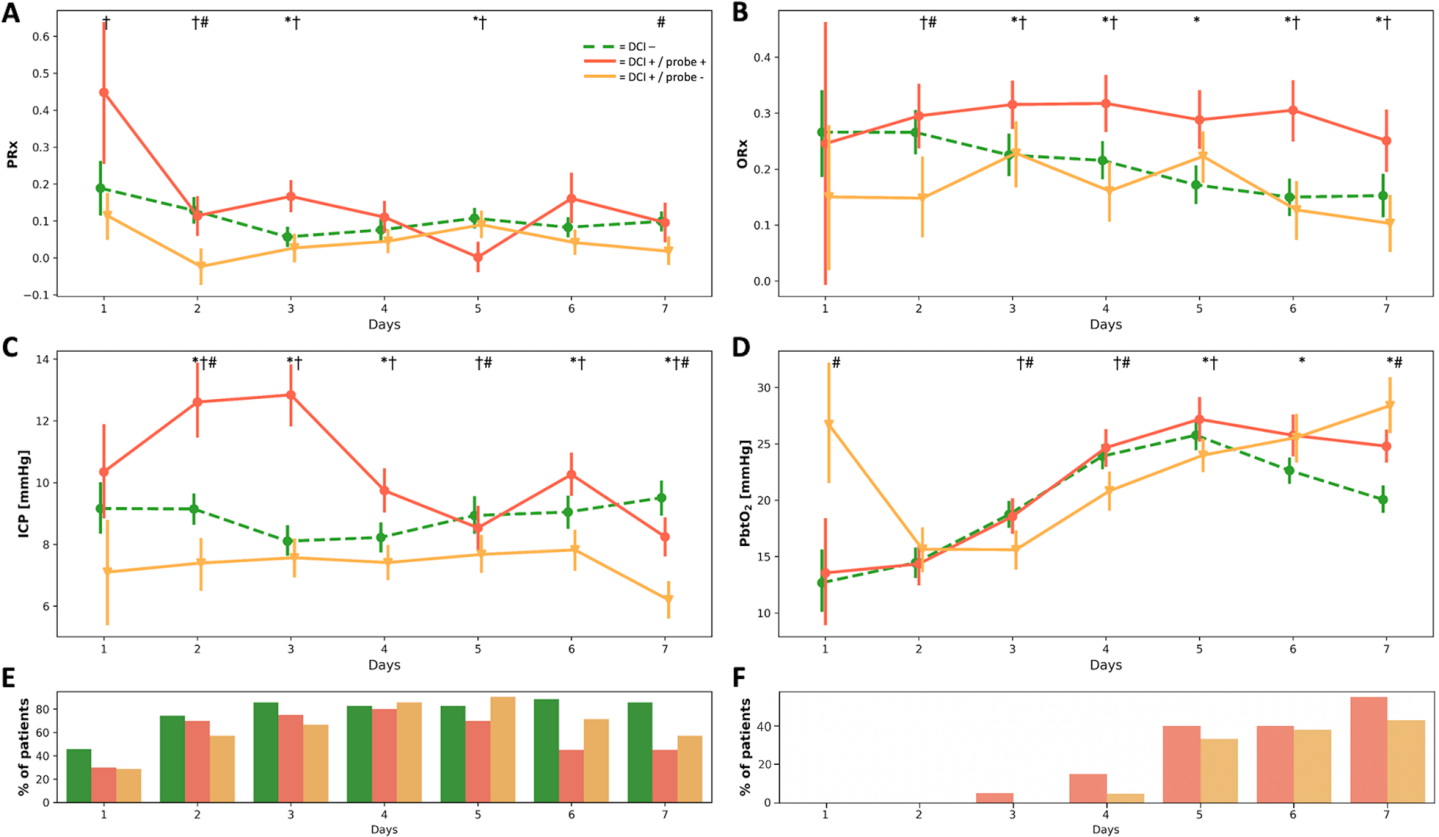
Daily values of neuromonitoring data from day of hemorrhage (Day one) until Day seven (average time of DCI), shown separately for the three subgroups (green: DCI−, orange: DCI + /probe−, red: DCI + /probe +). **A** Pressure Reactivity Index (PRx) showed significant differences during the first three days across all three groups, **B** Oxygen Reactivity Index (ORx) in the DCI + /probe + group was significantly different and consistently higher than in the other groups, **C** Intracranial Pressure (ICP) is higher in DCI + /probe + group, especially in the first three days, **D** PbtO_2_ increased consistently in all groups between Days two and five, **E** Percentage of patients from each group that had data available for daily comparison, **F** Cumulative percentage of patients from the two DCI + groups that were excluded due to onset of DCI. Values (**A**–**D**) are expressed as mean (± 95% CI). * = DCI + /probe + versus DCI−, **†** = DCI + /probe + versus DCI + /probe−, **#** = DCI + /probe− versus DCI−

Averaged PRx over the total monitoring period did not differentiate patients from the three groups (DCI− : 0.08 ± 0.17, DCI + /probe + : 0.12 ± 0.17, DCI + /probe− : 0.06 ± 0.20, *p* = 0.35). When stratified by days after hemorrhage, PRx was highest on the first day after hemorrhage in all three groups (DCI− : 0.19 ± 0.35; DCI + /probe + : 0.45 ± 0.39, DCI + /probe− : 0.11 ± 0.23, *p* < 0.05; Fig. 4A). PRx was highest on days 1–3 in DCI + /probe + patients, who also had the highest ICP values on those days. The following days, PRx converged at lower values in all groups.

Averaged ORx over the total monitoring period was significantly higher in patients with a probe in the hypoperfused area (DCI-: 0.20 ± 0.14, DCI + /probe + : 0.28 ± 0.13, DCI + /probe− : 0.18 ± 0.15, *p* < 0.05). Initial ORx from DCI + /probe + patients was 0.25 ± 0.41, increased to a maximum of 0.32 ± 0.37 on day 3 and remained elevated around this level throughout day 7 (Fig. 4B). DCI + /probe−  patients had significantly lower ORx values than DCI + /probe + patients from day 2 onward (DCI + /probe− : 0.15 ± 0.37 vs. DCI + /probe + : 0.30 ± 0.39, *p* < 0.001). ORx values of DCI- patients were highest on day 1 (0.27 ± 0.36) and decreased continuously.

## Discussion

Autoregulation-targeted treatment is under investigation as an alternative to the traditional, fixed pressure-targets after acute neurological injury [18, 27, 28]. The success of such strategies will depend on the how well they can be tailored to the individual patient to reach best possible efficacy, which requires attention to the specific disease properties such as DCI after aSAH. PRx has been mostly validated in TBI patients [8, 11]; however, whether the PRx is most useful as a target parameter in aSAH patients as opposed to other autoregulation indices such as the ORx remains unclear.

The assumption behind PRx is that fluctuations of blood pressure should trigger adjustments of cerebral vessel tone with vasoconstriction or vasodilation to maintain constant CBF, resulting in changes of ICP due to the spatial limitation of the neurocranium [29, 30]. PRx therefore represents the myogenic response, which is what is classically understood as ‘cerebral autoregulation’ [6]. ORx has been credited with the ability to quantify cerebral autoregulation as well [16, 17, 31], although it is less clear which components of autoregulation would be measured. ORx is based on fluctuations of PbtO_2_ as a response to changes in CPP and their relationship is probably not directly dependent on the pressure–volume curve. It may rather be a surrogate marker of cerebral perfusion, confounded by several factors such as oxygen consumption, hemoglobin, pH, PaCO_2_, PaO_2_ and others [32].

We found that both PRx and ICP were higher in patients whose probe was in a later hypoperfused area only during the first three days after hemorrhage, but both parameters returned to normal values afterward. It was previously suggested in TBI patients that PRx reflects autoregulation best, if the ICP is at least moderately elevated, because there is not a large volume reserve buffering fluctuations of vessel diameter [29]. Our data support this hypothesis: In all groups, PRx was higher when ICP was higher than on other days, and even more so in the DCI + /probe + group with the highest ICP in absolute numbers. Both could be a result of early brain injury and be associated with the later development of DCI [4, 12]. The following return of PRx to normal values could mean that autoregulation was also normal. However, perhaps PRx was simply not able to detect potentially dysfunctional autoregulation because ICP was lower and any blood-pressure passive changes in vessel volume would be buffered by the intracranial volume reserve. The ORx was also different in the early phase with higher ICP, but less pronounced than the PRx. In contrast to PRx, ORx did not lose this discriminating potential over time with lower ICP. If the probe was located outside of the hypoperfused area or if the patient did not develop DCI, ORx was significantly lower on almost all days with decreasing tendency as a sign of neuronal recovery after initial bleeding.

We believe that there are meaningful differences between PRx and ORx and their ability to discriminate the later development of DCI: PRx displays the dynamic of ICP, while ORx may differentiate DCI more uncoupled from ICP. TBI patients frequently suffer from elevated ICP, which may account for the good correlation of PRx with patient outcome after TBI [33]. However, although clinical outcome after aSAH is similarly associated with elevated ICP [14], DCI primarily occurs due to reduced cerebral perfusion, during oftentimes normal ICP measurements. PRx does not reflect changes in local perfusion equally and may therefore be less sensitive to differentiate patients with and without DCI much before it is diagnosed. We previously found a significant negative deviation of CPP from CPPopt together with a worsening of PRx a few hours before the diagnosis of DCI [20]. Thus, the full development of yet untreated DCI may be necessary for PRx to detect it, while the ORx could be more sensitive to less pronounced perfusion changes earlier on. PbtO_2_ was actually higher in those patients with the probe located within the DCI territory on most days following hemorrhage, and lower in DCI patients whose probe was not in an affected area. Thus, the ORx showed the most potential to discriminate early on which patients later developed DCI as opposed to PRx and the PbtO_2_ signal alone when probe location was considered retrospectively.

PRx and ORx values were not correlated in our cohort, supporting the notion that PRx and ORx measure inherently different processes. Jaeger et al. and Owen et al. found that ORx was associated with delayed cerebral infarctions and that PRx and ORx correlated, but these results could not be reproduced in other studies [13, 16, 31, 34, 35]. These ambiguous results may be partially explained by different calculation methods (averaging of raw data and length of moving correlation window), and it has been shown that PbtO_2_ and, to a lesser extent, ORx can differ depending on the probe technology [36]. Gaasch et al. concluded in their study that there was no association of ORx and development of DCI or patient outcome [12], but the location of the probe versus that of DCI-related hypoperfusion was not considered. Having retrospective knowledge of which territories were affected by DCI, we found that ORx—if measured in the right territory—was worse than in other patients.

For the design of autoregulation-targeted treatment strategies after aSAH, it is an important question whether other autoregulation indices are more sensitive and can detect failure of autoregulation due to hypoperfusion earlier than the PRx, allowing to take countermeasures earlier. Evidence on the regionality of autoregulation in the brain is largely lacking [37], but our results using ORx indicate that autoregulation could be poorer in territories affected by DCI. Using the ORx of a probe located in this region could be a highly meaningful therapeutic target, as it might represent the area most at risk, while it might be less important at that point to target areas that are still well-compensated. Due to this particular strength, ORx appears to be an interesting additional index to investigate in goal-directed therapy specifically for patients with aSAH. Both PRx and ORx may not capture all autoregulation deficits universally, the PRx due to the pressure–volume relationship and the ORx due to the regionality of the information. Non-invasive autoregulation measures, for example based on NIRS or TCD, are available, but have limitations of their own. Future investigations will need to determine which one or combination of autoregulation indices has most power to detect DCI and serve as a basis for autoregulation-targeted treatment.

### Limitations

This is a retrospective analysis of prospectively collected data, which can be associated with unknown bias at different steps of the analysis. Even though we present data from the largest aSAH cohort investigating ORx in relation to DCI, subgroup analysis may lack statistical power. PRx and ORx measurements were only available in patients that were equipped with an ICP/PbtO_2_-probe and expected to be at the highest risk of DCI, potentially introducing a certain selection bias. Lastly, our DCI diagnosis includes a definition based on CT perfusion deficit accounting for a higher than usual DCI rate [38]. Although this definition differs from others investigating DCI [39], most pathophysiological mechanisms that lead to DCI are assumed to result in hypoperfusion, therefore enabling an imaging diagnosis in comatose patients not qualifying for the definition of clinical deterioration by Vergouwen et al. and analysis of probe location [39].

## Conclusion

As opposed to PRx, ORx may not reflect the myogenic reaction as a part of cerebral autoregulation, and should not be used interchangeably with PRx, but rather as an adjunct monitor for cerebral perfusion regulation. While PbtO_2_ reflects more than just cerebral blood flow, perhaps its correlation with CPP enhances the CBF signal for the early detection of local perfusion disturbances associated with DCI. Further studies should investigate the most promising constellation of autoregulation indices to be used in autoregulation-targeted treatment investigations after aSAH.

## Data Availability

The datasets used and/or analyzed during the current study are available from the corresponding author on reasonable request.

## Abbreviations

aSAH: Aneurysmal subarachnoid hemorrhage
CA: Cerebral autoregulation
CBF: Cerebra blood flow
CPP: Cerebral perfusion pressure
CPPopt: Optimal cerebral perfusion pressure
CTP: CT perfusion imaging
ICP: Intracranial pressure
DCI: Delayed cerebral ischemia
HH: Hunt and Hess
MAP: Mean arterial pressure
mFS: Modified Fisher scale
mRS: Modified Rankin scale
ORx: Oxygen reactivity index
PbtO_2_: Brain tissue oxygenation
PRx: Pressure reactivity index
TCD: Transcranial Doppler

## References

[1] Dorhout Mees SM, Kerr RS, Rinkel GJ, Algra A, Molyneux AJ (2012). Occurrence and impact of delayed cerebral ischemia after coiling and after clipping in the International Subarachnoid Aneurysm Trial (ISAT). J Neurol.

[2] Macdonald RL, Higashida RT, Keller E, Mayer SA, Molyneux A, Raabe A (2011). Clazosentan, an endothelin receptor antagonist, in patients with aneurysmal subarachnoid haemorrhage undergoing surgical clipping: a randomised, double-blind, placebo-controlled phase 3 trial (CONSCIOUS-2). Lancet Neurol.

[3] Geraghty JR, Testai FD (2017). Delayed cerebral ischemia after subarachnoid hemorrhage: beyond vasospasm and towards a multifactorial pathophysiology. Curr Atheroscler Rep.

[4] Budohoski KP, Czosnyka M, Kirkpatrick PJ, Smielewski P, Steiner LA, Pickard JD (2013). Clinical relevance of cerebral autoregulation following subarachnoid haemorrhage. Nat Rev Neurol.

[5] Dodd WS, Laurent D, Dumont AS, Hasan DM, Jabbour PM, Starke RM (2021). Pathophysiology of delayed cerebral ischemia after subarachnoid hemorrhage: a review. J Am Heart Assoc.

[6] Claassen J, Thijssen DHJ, Panerai RB, Faraci FM (2021). Regulation of cerebral blood flow in humans: physiology and clinical implications of autoregulation. Physiol Rev.

[7] Czosnyka M, Miller C (2014). Participants in the international multidisciplinary consensus conference on multimodality M. Monitoring of cerebral autoregulation. Neurocrit Care.

[8] Czosnyka M, Smielewski P, Kirkpatrick P, Piechnik S, Laing R, Pickard JD (1998). Continuous monitoring of cerebrovascular pressure-reactivity in head injury. Acta Neurochir Suppl.

[9] Steiner LA, Coles JP, Johnston AJ, Chatfield DA, Smielewski P, Fryer TD (2003). Assessment of cerebrovascular autoregulation in head-injured patients: a validation study. Stroke.

[10] Rivera-Lara L, Zorrilla-Vaca A, Geocadin R, Ziai W, Healy R, Thompson R (2017). Predictors of outcome with cerebral autoregulation monitoring: a systematic review and meta-analysis. Crit Care Med.

[11] Donnelly J, Aries MJ, Czosnyka M (2015). Further understanding of cerebral autoregulation at the bedside: possible implications for future therapy. Expert Rev Neurother.

[12] Gaasch M, Schiefecker AJ, Kofler M, Beer R, Rass V, Pfausler B (2018). Cerebral autoregulation in the prediction of delayed cerebral ischemia and clinical outcome in poor-grade aneurysmal subarachnoid hemorrhage patients. Crit Care Med.

[13] Barth M, Woitzik J, Weiss C, Muench E, Diepers M, Schmiedek P (2010). Correlation of clinical outcome with pressure-, oxygen-, and flow-related indices of cerebrovascular reactivity in patients following aneurysmal SAH. Neurocrit Care.

[14] Svedung Wettervik T, Howells T, Lewén A, Ronne-Engström E, Enblad P (2021). Temporal dynamics of ICP, CPP, PRx, and CPPopt in high-grade aneurysmal subarachnoid hemorrhage and the relation to clinical outcome. Neurocrit Care.

[15] Johnson U, Engquist H, Howells T, Nilsson P, Ronne-Engstrom E, Lewen A (2016). Bedside xenon-CT shows lower CBF in SAH patients with impaired CBF pressure autoregulation as defined by pressure reactivity index (PRx). Neurocrit Care.

[16] Jaeger M, Schuhmann MU, Soehle M, Nagel C, Meixensberger J (2007). Continuous monitoring of cerebrovascular autoregulation after subarachnoid hemorrhage by brain tissue oxygen pressure reactivity and its relation to delayed cerebral infarction. Stroke.

[17] Jaeger M, Soehle M, Schuhmann MU, Meixensberger J (2012). Clinical significance of impaired cerebrovascular autoregulation after severe aneurysmal subarachnoid hemorrhage. Stroke.

[18] Tas J, Beqiri E, van Kaam RC, Czosnyka M, Donnelly J, Haeren RH (2021). Targeting autoregulation-guided cerebral perfusion pressure after traumatic brain injury (COGiTATE): a feasibility randomized controlled clinical trial. J Neurotrauma.

[19] Megjhani M, Weiss M, Ford J, Terilli K, Kastenholz N, Nametz D (2022). Optimal cerebral perfusion pressure and brain tissue oxygen in aneurysmal subarachnoid hemorrhage. Stroke.

[20] Weiss M, Albanna W, Conzen C, Megjhani M, Tas J, Seyfried K (2022). Optimal cerebral perfusion pressure during delayed cerebral ischemia after aneurysmal subarachnoid hemorrhage. Crit Care Med.

[21] Rijsdijk M, van der Schaaf IC, Velthuis BK, Wermer MJ, Rinkel GJ (2008). Global and focal cerebral perfusion after aneurysmal subarachnoid hemorrhage in relation with delayed cerebral ischemia. Neuroradiology.

[22] von Elm E, Altman DG, Egger M, Pocock SJ, Gotzsche PC, Vandenbroucke JP (2007). Strengthening the reporting of observational studies in epidemiology (STROBE) statement: guidelines for reporting observational studies. BMJ.

[23] Weiss M, Albanna W, Conzen-Dilger C, Kastenholz N, Seyfried K, Ridwan H (2022). Intraarterial nimodipine versus induced hypertension for delayed cerebral ischemia: a modified treatment protocol. Stroke.

[24] Vergouwen MD, Vermeulen M, van Gijn J, Rinkel GJ, Wijdicks EF, Muizelaar JP (2010). Definition of delayed cerebral ischemia after aneurysmal subarachnoid hemorrhage as an outcome event in clinical trials and observational studies: proposal of a multidisciplinary research group. Stroke.

[25] Czosnyka M, Smielewski P, Kirkpatrick P, Laing RJ, Menon D, Pickard JD (1997). Continuous assessment of the cerebral vasomotor reactivity in head injury. Neurosurgery.

[26] Jaeger M, Schuhmann MU, Soehle M, Meixensberger J (2006). Continuous assessment of cerebrovascular autoregulation after traumatic brain injury using brain tissue oxygen pressure reactivity. Crit Care Med.

[27] Needham E, McFadyen C, Newcombe V, Synnot AJ, Czosnyka M, Menon D (2017). Cerebral perfusion pressure targets individualized to pressure-reactivity index in moderate to severe traumatic brain injury: a systematic review. J Neurotrauma.

[28] Donnelly J, Czosnyka M, Adams H, Robba C, Steiner LA, Cardim D (2017). Individualizing thresholds of cerebral perfusion pressure using estimated limits of autoregulation. Crit Care Med.

[29] Aries MJ, Czosnyka M, Budohoski KP, Kolias AG, Radolovich DK, Lavinio A (2012). Continuous monitoring of cerebrovascular reactivity using pulse waveform of intracranial pressure. Neurocrit Care.

[30] Czosnyka M, Brady K, Reinhard M, Smielewski P, Steiner LA (2009). Monitoring of cerebrovascular autoregulation: facts, myths, and missing links. Neurocrit Care.

[31] Owen B, Vangala A, Fritch C, Alsarah AA, Jones T, Davis H (2022). Cerebral autoregulation correlation with outcomes and spreading depolarization in aneurysmal subarachnoid hemorrhage. Stroke.

[32] Nordstrom CH, Koskinen LO, Olivecrona M (2017). Aspects on the physiological and biochemical foundations of neurocritical care. Front Neurol.

[33] Cabella B, Donnelly J, Cardim D, Liu X, Cabeleira M, Smielewski P (2017). An association between ICP-derived data and outcome in TBI patients: the role of sample size. Neurocrit Care.

[34] Andresen M, Donnelly J, Aries M, Juhler M, Menon D, Hutchinson P (2018). Further controversies about brain tissue oxygenation pressure-reactivity after traumatic brain injury. Neurocrit Care.

[35] Zeiler FA, Donnelly J, Menon DK, Smielewski P, Zweifel C, Brady K (2017). Continuous autoregulatory indices derived from multi-modal monitoring: each one is not like the other. J Neurotrauma.

[36] Dengler J, Frenzel C, Vajkoczy P, Horn P, Wolf S (2013). The oxygen reactivity index and its relation to sensor technology in patients with severe brain lesions. Neurocrit Care.

[37] Hecht N, Schrammel M, Neumann K, Muller MM, Dreier JP, Vajkoczy P (2021). Perfusion-dependent cerebral autoregulation impairment in hemispheric stroke. Ann Neurol.

[38] Veldeman M, Albanna W, Weiss M, Conzen C, Schmidt TP, Schulze-Steinen H (2020). Invasive neuromonitoring with an extended definition of delayed cerebral ischemia is associated with improved outcome after poor-grade subarachnoid hemorrhage. J Neurosurg.

[39] Cremers CH, van der Schaaf IC, Wensink E, Greving JP, Rinkel GJ, Velthuis BK (2014). CT perfusion and delayed cerebral ischemia in aneurysmal subarachnoid hemorrhage: a systematic review and meta-analysis. J Cereb Blood Flow Metab.

